# A labeled spectral dataset with cassava disease occurrences using virus titre determination protocol

**DOI:** 10.1016/j.dib.2023.109387

**Published:** 2023-07-09

**Authors:** Godliver Owomugisha, Joyce Nakatumba-Nabende, Joshua Jeremy Dhikusooka, Estefania Taravera, Ephraim Nuwamanya, Ernest Mwebaze

**Affiliations:** a Faculty of Engineering, Busitema University, P. O. Box 236, Tororo, Uganda; b College of Computing & IS, Makerere University, P.O. Box 7062, Kampala, Uganda; c Faculty of Electrical Engineering, Data Management & Biometrics, University of Twente, P.O. Box 217 7500 AE, Enschede, the Netherlands; d National Crops Resources Research Institute, P.O Box 7084, Kampala, Uganda

**Keywords:** Spectral data protocol, Cassava diseases, Crop diagnosis, Smart agriculture, Early disease detection

## Abstract

In this work, we present a novel dataset composed of spectral data and images of cassava crops with and without diseases. Together with the description of the dataset, we describe the protocol to collect such data in a controlled environment and in an open field where pests are not controlled. Crop disease diagnosis has been done in the past through the analysis of plant images taken with a smartphone camera. However, in some cases, disease symptoms are not visible. Furthermore, for some cassava diseases, once symptoms have manifested on the aerial part of the plant, the root which is the edible part of the plant has been totally destroyed. The goal of collecting this multimodality of the crop disease is early intervention, following the hypothesis that diseased crops without visible symptoms can be detected using spectral information. We collected visible and near-infrared spectra captured from leaves infected with two common cassava diseases namely; Cassava Brown Streak Disease and Cassava Mosaic Disease, as well as from healthy plants. Together, we also captured leaf imagery data that corresponds to the spectral information. In our experiments, biochemical data is collected and taken as the ground truth. Finally, agricultural experts provided a disease score per plant leaf from 1 to 5, 1 representing healthy and 5 severely diseased. The process of disease monitoring and data collection took 19 and 15 consecutive weeks for screenhouse and open field, respectively, until disease symptoms were visibly seen by the human eye.

**Specifications Table**

**Table:** 

Subject	Agronomy and Crop Science, Artificial Intelligence.
Specific subject area	Cassava Brown Streak Disease (CBSD), Cassava Mosaic Disease (CMD), Spectral Data)
Type of data	Raw spectral data Leaf Imagery data Biochemical lab test data Visual expert scoring
How the data were acquired	Spectral data was collected using visible and near-infrared handheld spectrometer [1]. Leaf image data was captured using a smartphone camera of 12-megapixel. The biochemical data was extracted using a 96-well Real Time PCR instrument [2]. Finally, agricultural experts score plants as healthy or diseased based on visual symptoms that appear on the plants.
Data format	1. Raw data 2. Analyzed data
Description of data collection	The dataset is in two major groups: screenhouse and open field experiment, collected for 19 and 15 consecutive weeks respectively.
Data source location	The dataset is in two major groups: screenhouse and open field experiment, collected for 19 and 15 consecutive weeks respectively.
Data accessibility	Repository name: Harvard Dataverse Data identification number: doi:10.7910/DVN/R0KL7R Direct URL to data: https://dataverse.harvard.edu/dataset.xhtml?persistentId=doi:10.7910/DVN/R0KL7R
Related research article	Godliver Owomugisha, Ephraim Nuwamanya, John A. Quinn, Michael Biehl, and Ernest Mwebaze. 2020. Early detection of plant diseases using spectral data. In Proceedings of the 3rd International Conference on Applications of Intelligent Systems (APPIS 2020). Association for Computing Machinery, New York, NY, USA, Article 26, 1–6. https://doi.org/10.1145/3378184.3378222

## Value of the Data

• The collected samples are a contribution to the field of smart farming and agriculture, more specifically to the growing area of early disease detection in asymptomatic plants.
• The findings of this dataset will contribute to the breeding research to develop the best crop breeds.
• To our best knowledge, this is the first spectral dataset that is publicly available on cassava diseases, a crop that is highly researched on.
• This dataset will also pave the way for the same research in other crops in early disease detection.

## 1 Objective

The objective of this work is to provide a dataset of spectral information collected from cassava plants, which can be used to aid in the early detection of diseases in asymptomatic plants. The work aims to contribute to the growing area of smart farming and agriculture, specifically in the field of early disease detection in crops. The dataset will also contribute to breeding research, by helping to identify the best crop breeds. Additionally, the work aims to make this spectral dataset publicly available, which will make it the first dataset on cassava diseases. This dataset will not only benefit research on cassava diseases but also pave the way for research on early disease detection in other crops. Overall, the objective is to provide a valuable resource for researchers and professionals in the agricultural industry to improve crop yields and prevent losses due to diseases.

## 2 Data Description

We present a spectral dataset that was collected from healthy and infected plants in a controlled environment (screenhouse) and in a field setup. The screen house setup rules out the influence of other diseases, pests or severe weather conditions while in an open field, crops grow under a natural environment, also exposed to crop pests. The experiment was carried out in partnership with the National Crop Resources Research Institute (NaCRRI). The dataset is composed of two experiments: screenhouse and open field experiment. Each experiment contains the following data.

I. Spectral data. The spectrograms were acquired by a handheld spectrometer.
II. Leaf image data. For each plant, a corresponding image was acquired by a smartphone camera.
III. Biochemical data. Lab chemical data as a ground truth on disease propagation.
IV. Expert scoring. At the same, plants were scored every week by the agricultural experts on visual symptoms.

Each of the two main sub-folder contains other files. a) Folder holding raw spectra data. The CI-710 miniature leaf spectrometer device generates three files each time a record is captured. (i). csv file on calculations, csv on raw and calibrated spectra and .png graph. The optional calculations file includes formulas for different Pigments if specified. The several indexes can externally be calculated from the spectral bands [3]. (b). Folder containing pre-processed .csv files for the different categories. This data is an extraction of raw and calibrated data that was collected with a CI-710 miniature leaf spectrometer device. This data is labeled as shown in Table 3. The labels correspond to chemical lab tests, plant/leaf image and expert scoring. (c). Folder containing leaf imagery data in format .jpeg corresponding to raw spectra data in (a) above.

## 3 Experimental Design, Material and Methods

**Fig. 1:**
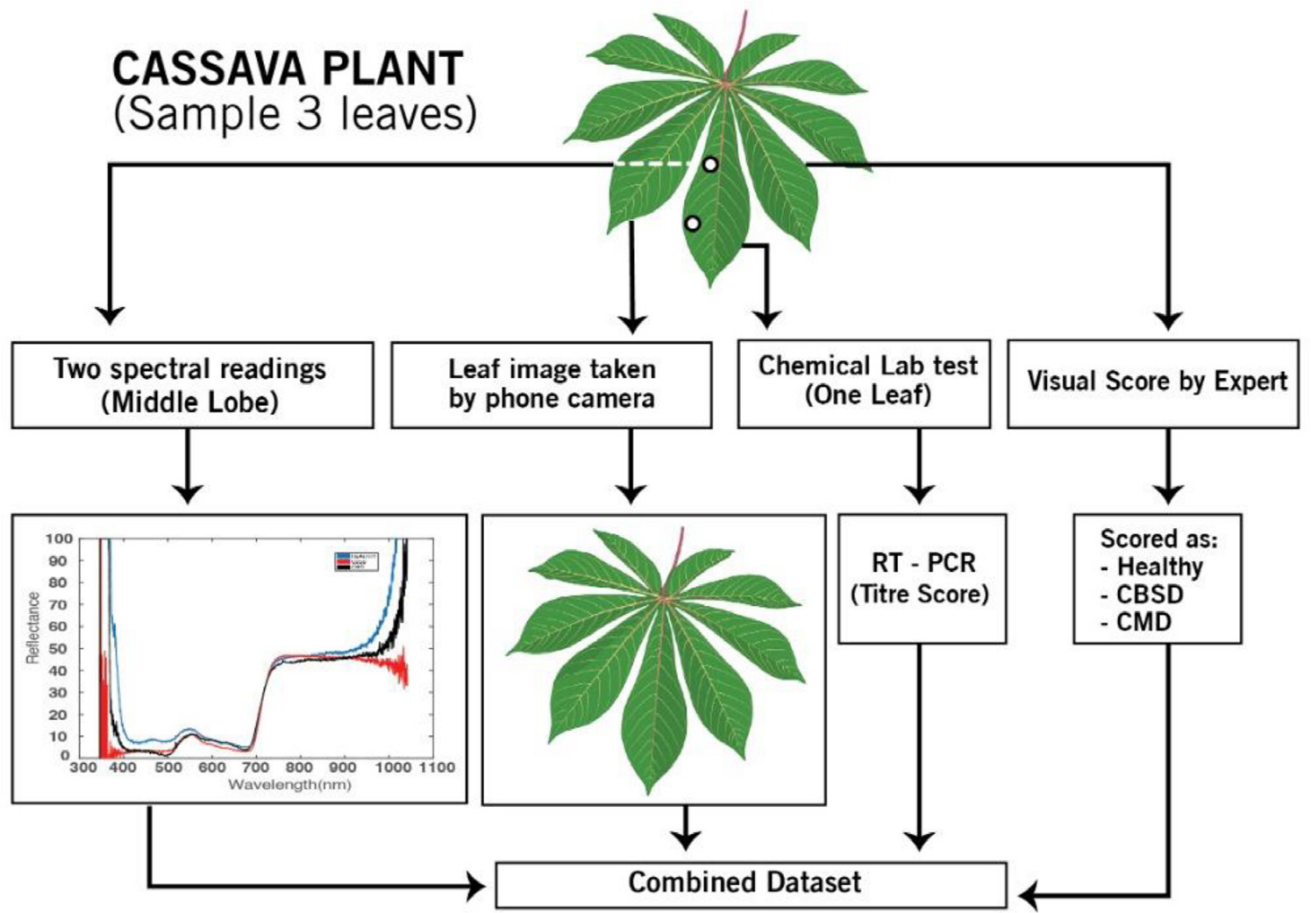
Illustration of the datasets generated from the experiment.

The experiment was conducted in a controlled screen house environment. The screen house setup rules out the influence of other diseases, pests or severe weather conditions while in an open field, crops grow under a natural environment, also exposed to crop pests. Healthy cassava stems were identified from clean cassava gardens by the agricultural experts. The plants were distributed across three varieties (NAROCass, TME14 and a local variety “Kwatamubale”). These varieties were chosen on the basis of being tolerant (NAROCass), susceptible (TME14, Kwatamubale). Initially, planting materials were thoroughly cleaned, which included the sterilization of the soil to ensure that no gaps led to disease transmission. At week four of growth, these plants were inoculated with CBSD and CMD diseases while maintaining a section as a healthy control. Spectral reading and chemical lab samples were collected for a period of 19 and 15 consecutive weeks of disease monitoring for the screenhouse and open field respectively. The data collection process ended when the disease symptoms started showing in some plants visibly seen by the human eye (Fig. 1).

## 4 Virus Titre Determination Protocol

### 4.1 Confirmation of CBSD Transmission

This protocol follows the initial work done in [4,5]. The DNA of a plant changes when it is infected by a particular virus. This can lead to the production of specific protein molecules derived from the pathogen causing the infection. The molecular-based and polymerase chain reaction (PCR-based) disease detection techniques are commonly used to confirm the transmission of viruses. Here, we discuss the procedures we follow to confirm the presence of the CBSD and CMD viruses in our study of cassava plants grown in controlled environments, i.e. in screen houses. The diagnosis involves the step by step examination of infection in the plants due to any of the viruses under study, i.e. confirming the presence or absence of the virus titre. The following key steps were taken during diagnosis of the disease following:

– Special care was taken to observe the disease in the field, determine which plants were affected and from which test varieties to establish disease incidence.
– Symptoms were noted to determine the presence of the disease in each of the plants observed.
– The persistence of the observed symptoms was determined by periodic observations of infected leaves and stems.
– Leaf samples were collected from all test plants following the relevant protocols and sent to the laboratory for confirmation.

Preparation of tissue samples for PCR/RT-PCR assay included the following:

– Leaf tissue was ground at a ratio of 1:20 (w/v) in a buffer. To sample grinding we used sterile motor and pestle.
– The extract was aliquoted and stored at 20/-80*^◦^*C for a later analysis.
– Pl of the extract from step 1 was picked after thawing and mixed with 25 Pl of GES buffer.
– The sample was vortexed and heat denatured at 95*^◦^*C for 10 min in a waterbath. The tubes were then placed on ice for 5 min and thereafter the rt-PCR reactions were undertaken following the procedure as described in the section below.

### 4.2 Real-Time Polymerase Chain Reaction (RT-PCR)

The reactions were prepared in a 96 well plate and analyzed with RT-PCR to detect the two viruses CBSV and UCBSV. As a control, a COX assay was also carried out. COX is a widely used housekeeping gene for normalizing cycle threshold (Ct) values. The COX assay was performed to see if there was cDNA in the samples. Three master mixes were made (CBSV, UCBSV and COX) with the final concentration of 10 ul 2x Sso advanced Universal SYBR green super mix, 1 ul of 10 pmol/ul forward primer, 1 ul of 10 pmol/ul reverse primer, 6 ul of nuclease free water and 2 ul of cDNA per reaction. The Real-Time amplification program was set; initial denaturation 95*^◦^*C for 30 min followed by 40 cycles of Denaturation at 95*^◦^* C for 10 sec and annealing at 56*^◦^* C for 30 sec. cDNA from CBSV- and UCBSV- infected plants were used as positive controls. A negative control with all the reagents and sterile distilled water instead of cDNA was used.

## 5 Data Preparation

### 5.1 Spectral Data

We extract raw spectral and calibrated data for each plant generated by the spectrometer device. This data comes in a wavelength of 1042 nms with 3652 features shown in Table 2. On this data, we append RT-PCR data provided by the Lab chemists that corresponds to each spectra. Together, leaf image and the scoring by the agricultural expert are appended as seen in Table 3.

### 5.2 Cleaning and Annotation

The data cleaning and annotation process involves: checking missing values, aggregating files and ensuring that all spectral data points have corresponding values. Sources of noncontinuous data could arise if a plant dried up. However, this process is able to detect that and such plants were eliminated from the dataset (Table 1).

**Table 1:** Data summary for different categories.

Spectral Data	Screenhouse	Open field	Corresponding data types
Reading 1	4080	3780	Biochemical data, Expert Score, Image data
Reading 2	4080	3780	Biochemical data, Expert Score, Image data

**Table 2:** Projection of the top 9 spectral data points with 3652 features.

0	19.91701	2.259887	1.167315	0	0	0	0	0
68.5259	81.21827	100	94.4	100	100	100	100	100
15.53785	0	0	0	56.25	100	96.19048	100	82.42424
91.23506	94.41624	65.90909	74.4	63.88889	68.86792	82.85714	94.52055	58.18182
100	100	100	100	100	100	43.80952	60.27397	72.72727
98.80478	100	100	100	100	100	100	100	100
79.68127	86.80203	52.27273	23.2	84.72222	100	91.42857	100	100
78.08765	65.98985	58.33333	100	96.52778	100	100	100	30.30303
94.42231	94.92386	87.87879	100	100	100	100	100	100

### 5.3 Labeling

At the start of data collection, all plants are tagged using our standard method e.g. A 1 HLT 1 R a 1g. This naming format applies to all the data types (spectral data, image data, Biochemical, scoring by experts) to uniquely identify a data point. This data is also cross checked on a weekly basis to ensure it is consistent. The Label A 1 HLT 1 R a 1g is translated as: A stands for variety where there are three varieties in our case (A, B and C). The number 1 means the week 1 of data collection, HLT is the control class which means healthy. The variable can be substituted for CMD or CBSD for diseased classes. After the class, variable 1 stands for plant number, the next variable which is R stands for reflectance mode. After the mode, the variable a stands for leaf, data is collected from three leaves per plant, that is from leaf a, b and c. The last variables “1g” are treated as constants or placeholders. After data collection, data points collected from all weeks are combined into one .csv file. Labels are appended, occupying the extreme last columns as shown in Table 3.

**Table 3:** Corresponding labels for the above spectral data points. Class represents a disease class, followed by the week the data was collected, variety type, plant ID, leaf number, image label corresponding to the actual file in the image folder, expert score, chemistry lab test. Class 1 means Healthy, 2 means CBSD and 3 means CMD.

class	week	variety	plant	leaf	image_label	image_name	expert_score	chemistry test
1	1	2	2	1	B1HLT1Ra1g	1617009756154.jpg	1	-0.4074
1	1	2	2	2	B1HLT2Ra1g	1617010171438.jpg	1	-0.4074
1	1	2	2	3	B1HLT2Rc1g	1617010380406.jpg	1	-0.4074
1	1	2	3	1	B1HLT3Ra1g	1617010491023.jpg	1	-0.3598
1	1	2	3	2	B1HLT3Rb1g	1617010551868.jpg	1	-0.3598
1	1	2	3	3	B1HLT3Rc1g	1617010641557.jpg	1	-0.3598
1	1	2	4	1	B1HLT4Ra1g	1617010721416.jpg	1	-0.4123
1	1	2	4	2	B1HLT4Rb1g	1617010792851.jpg	1	-0.4123
1	1	2	4	3	B1HLT4RC1g	1617010854553.jpg	1	-0.4123

## Ethics Statements

The study does not involve experiments on humans or animals.

## CRediT authorship contribution statement

**Godliver Owomugisha:** Writing – review & editing, Conceptualization, Methodology. **Joyce Nakatumba-Nabende:** Conceptualization, Methodology. **Joshua Jeremy Dhikusooka:** Writing – original draft. **Estefania Taravera:** Writing – review & editing. **Ephraim Nuwamanya:** Data curation. **Ernest Mwebaze:** Conceptualization, Methodology.

## Declaration of Competing Interest

The authors declare that the research was conducted in the absence of any commercial or financial relationships that could be construed as a potential conflict of interest.

## Data Availability

Cassava Spectral and Image Dataset (Original data) (Dataverse).
